# Human neural network activity reacts to gravity changes *in vitro*

**DOI:** 10.3389/fnins.2023.1085282

**Published:** 2023-03-08

**Authors:** Johannes Striebel, Laura Kalinski, Maximilian Sturm, Nils Drouvé, Stefan Peters, Yannick Lichterfeld, Rouhollah Habibey, Jens Hauslage, Sherif El Sheikh, Volker Busskamp, Christian Liemersdorf

**Affiliations:** ^1^Department of Ophthalmology, Medical Faculty, University of Bonn, Bonn, Germany; ^2^Department of Gravitational Biology, Institute of Aerospace Medicine, German Aerospace Center, Cologne, Germany; ^3^Department of Applied Sciences, Cologne University of Applied Sciences, Leverkusen, Germany

**Keywords:** multi-electrode array (MEA), microgravity, hypergravity, neural network, human induced pluripotent stem cell (hiPSC)-derived neurons, drop tower, electrophysiology, iNGN

## Abstract

During spaceflight, humans experience a variety of physiological changes due to deviations from familiar earth conditions. Specifically, the lack of gravity is responsible for many effects observed in returning astronauts. These impairments can include structural as well as functional changes of the brain and a decline in cognitive performance. However, the underlying physiological mechanisms remain elusive. Alterations in neuronal activity play a central role in mental disorders and altered neuronal transmission may also lead to diminished human performance in space. Thus, understanding the influence of altered gravity at the cellular and network level is of high importance. Previous electrophysiological experiments using patch clamp techniques and calcium indicators have shown that neuronal activity is influenced by altered gravity. By using multi-electrode array (MEA) technology, we advanced the electrophysiological investigation covering single-cell to network level responses during exposure to decreased (micro-) or increased (hyper-) gravity conditions. We continuously recorded in real-time the spontaneous activity of human induced pluripotent stem cell (hiPSC)-derived neural networks *in vitro*. The MEA device was integrated into a custom-built environmental chamber to expose the system with neuronal cultures to up to 6 g of hypergravity on the Short-Arm Human Centrifuge at the DLR Cologne, Germany. The flexibility of the experimental hardware set-up facilitated additional MEA electrophysiology experiments under 4.7 s of high-quality microgravity (10^–6^ to 10^–5^ g) in the Bremen drop tower, Germany. Hypergravity led to significant changes in activity. During the microgravity phase, the mean action potential frequency across the neural networks was significantly enhanced, whereas different subgroups of neurons showed distinct behaviors, such as increased or decreased firing activity. Our data clearly demonstrate that gravity as an environmental stimulus triggers changes in neuronal activity. Neuronal networks especially reacted to acute changes in mechanical loading (hypergravity) or de-loading (microgravity). The current study clearly shows the gravity-dependent response of neuronal networks endorsing the importance of further investigations of neuronal activity and its adaptive responses to micro- and hypergravity. Our approach provided the basis for the identification of responsible mechanisms and the development of countermeasures with potential implications on manned space missions.

## 1. Introduction

In space, astronauts are confronted with challenging environmental conditions, such as exposure to hyper- as well as microgravity and higher radiation doses. These stimuli are critical to human health and lead to deteriorated muscle performance (LeBlanc et al., 2000), reduced immune function (Crucian et al., 2000; Paulsen et al., 2010), loss of bone mass (Smith et al., 1999; Sibonga, 2013) but also affect brain health and cognitive performance (Clément, 2011; Roberts et al., 2017; Van Ombergen et al., 2017, 2018; Wollseiffen et al., 2019; Popova et al., 2020).

Long-standing efforts have been implemented to expand human explorative spaceflight missions, while simultaneously diminishing the impact on astronaut health. Nevertheless, little is known about the effects of altered gravity, the most prominent environmental cue in space conditions, on neuronal function. However, spaceflight has impacts on brain structure and function, and potential consequences are spatial disorientation, visual and motor dysfunction, impaired focus, and a general decline in cognitive task performance (Kanas and Manzey, 2008; Garrett-Bakelman et al., 2019; Roy-O’Reilly et al., 2021). By comparing magnetic resonance imaging (MRI) scans of astronauts’ brains before and after space missions, structural changes in grey matter along with decreased neuronal plasticity were detected in individuals spending either 2 weeks or 12 months on the International Space Station (ISS) (Demertzi et al., 2016; Van Ombergen et al., 2017). Anatomical changes have been observed as well (Roberts et al., 2017; Van Ombergen et al., 2018). Therefore, the risk of long-term exposure to altered gravity resulting in long-lasting neurodegenerative events after return to Earth cannot be excluded (Roberts et al., 2020). Consequently, the impact of varying gravity environments on cellular mechanisms of neurons leading to such changes needs to be explored.

Several approaches have been applied to investigate the response of various cell types to simulated micro- or hypergravity (Galimberti et al., 2006; Maier et al., 2015). For example, in neurons, abnormalities in the cytoskeletal network, such as microtubule and actin networks, have been observed under altered gravity conditions (Rösner et al., 2006). These morphological alterations manifest changes in important neuron-specific cellular parameters like neurite growth, neurite area, and microtubule filament distribution (Pani et al., 2013; Genchi et al., 2015). Cytoskeletal integrity seems to be crucial for maintenance of cellular functions. Astrocytes also react to hypergravity with changes in morphology, migration, and reduced reactivity that were also related to altered cytoskeletal dynamics (Lichterfeld et al., 2022). Given that neuronal cytoskeletal changes are involved in several developmental phases, alteration in the establishment, maintenance, or retraction of synaptic contacts can be assumed. Therefore, the most crucial process of neuronal transmission is likely influenced, too. Thus, identifying the impact and understanding the risks that arise from altered neuronal signal transmission under micro- and hypergravity conditions is of fundamental importance. Moreover, functional electrophysiological output is a central part of neuronal health and lack thereof is a hallmark of neurodegenerative or neurological disorders (Chong et al., 2011; Babiloni et al., 2020).

Currently, little is known about prolonged effects of altered gravity on neuronal function at the cellular and especially network level. However, previous experiments with patch-clamping and extracellular electrodes on earthworm and rat neurons in the drop tower (ZARM, Bremen, Germany) and on parabolic flights have shown that propagation velocity of action potentials (APs) respond in a gravity-dependent fashion (Meissner and Hanke, 2002, 2005). This observation has led to the hypothesis that microgravity exposure leads to an increase in the frequency of spontaneous activity. This could be a result of increased lipid membrane fluidity under microgravity as it was observed in neurons under parabolic flight conditions (Sieber et al., 2014) and in plain lipid membranes during 6 min of microgravity in a sounding rocket experiment (Kohn et al., 2017; Kohn and Hauslage, 2019). In neurons, lower gravity levels would thus lead to increased excitability of the membrane due to a higher open state probability of the ion channels, lowering the threshold for evoking APs. Cytosolic calcium concentrations were used previously to visualize neuronal activity changes in SH-SY5Y neuroblastoma cells. These cells were exposed to simulated microgravity on the clinostat, as well as to parabolic flights, that provided a repeated and alternating change of micro- and hypergravity phases of approximately 22 s. This resulted in a change in calcium permeability of the membrane and indicates a response to gravity changes (Hauslage et al., 2016). Peripheral nerve stimulation in human subjects also indicated altered characteristics of synaptic and axonal nerve conductivity and excitability (Ritzmann et al., 2017).

While these findings emphasize the need for more detailed research, a set-up for accurate measurements of electrophysiological activity of live neurons and whole neural networks under altered gravity conditions is difficult to realize. Gravity research platforms include large facilities with limited access, such as drop towers, parabolic flights with airplanes or sounding rockets to achieve microgravity conditions on Earth or various kinds of centrifuges to induce hypergravity as a form of enhanced mechanical loading. These facilities pose demands on the experiment hardware like size restrictions, structural integrity, and limited availability of support systems like electricity. Additionally, the hardware needs to satisfy the demands posed by the live biological samples.

Further, a technique was needed for accurate measurements of electrophysiological activity of cultured neuronal networks under the extreme mechanical loading conditions of altered gravity. Multi-electrode array (MEA) technology enables the investigation of electrophysiological activity of neural networks in real time over extended time periods. Neurons are grown on a glass chip inlaid with a grid of microelectrodes. When excited, neurons create a difference in electric potential between the inner and the outer side of the cell membrane, which is detected by the electrodes. In contrast to patch-clamp assays, MEA technology allows for non-invasive extracellular electrophysiology measurements and can be used for assessment of functional connectivity of multiple neurons over long periods of time. Additionally, the system is flexible and simple to operate making it ideal for implementation on different and technologically highly demanding gravity research platforms. For instance, studies using maize roots mounted atop a multi-electrode array chip have been carried out under micro- and hypergravity and have shown that AP duration and propagation velocity were significantly altered by both gravity stimuli (Masi et al., 2015).

Here, we report the measurement of the electrophysiological behavior of functional neural networks derived from human induced pluripotent stem cells (hiPSCs) under the influence of altered gravity. Post-mitotic human neurons can be generated within 4 days by the overexpression of two transcription factors (TFs), Neurogenin-1 and Neurogenin-2 with an efficiency of more than 90% (Busskamp et al., 2014). The TFs are driven from an inducible TetOn promoter and the entire cassette is stably integrated into hiPSCs. This well-established iNGN cell line represents a relatively homogeneous population of excitatory neurons (Busskamp et al., 2014; Lam et al., 2017; Kutsche et al., 2018). iNGN-derived neurons require an additional time for functional maturation (Lam et al., 2017; Schmieder et al., 2022). During this maturation phase a connected network of neurons develops, network bursts arise, and a GABAergic system evolves, resembling human brain development (Habibey et al., 2022). In the first 4 days of induction, an increase in the expression of glutamatergic genes was measured (Busskamp et al., 2014). At 30 days post induction (dpi), about 2.3% of neurons have been shown to be GABAergic (Lu et al., 2019). This fraction increases over time and recent results suggest an increase in the number of inhibitory neurons after 60 dpi (Habibey et al., 2022). More detailed functional characterization of long-term development of iNGN networks has shown that the majority of iNGN neurons have functional synapses after 21 days and functional AMPA/KA receptors are present in iNGN neurons as confirmed by immunohistochemistry and patch-clamp experiments (Lam et al., 2017). These iNGN cells can serve as an experimental platform for basic and biomedical investigation providing entire human neural networks *in vitro* instead of single, isolated cells.

A custom-built environmental chamber allowed us to perform the experiments in various environments, such as the ZARM drop tower in Bremen, Germany, and the Short Arm Human Centrifuge (SAHC) of the German Aerospace Center (DLR) in Cologne, Germany. The set-up maintains constant physiological conditions for the cells over several hours and shields them from external influences such as electromagnetic disturbances or pressure changes. Employing free fall in the drop tower and centrifugation in the SAHC, we subjected human neural networks to 4.7 s of 10^–6^–10^–5^ g (μg, microgravity) and 5 min of hypergravity (up to 6 g). Our measurements revealed changes in electrophysiological activity following these interventions. More detailed analysis demonstrated complex network activity responses to the stimulus, with neuronal subgroups reacting differently to the change in gravity and thus mechanical loading.

## 2. Materials and methods

### 2.1. Cell culture

HiPSCs were used to establish iNGN cells (kind gift of George Church). Upon overexpression of Neurogenin-1 and Neurogenin-2 under the TetOn inducible promoter system, cells differentiate into post-mitotic, bipolar neurons (Busskamp et al., 2014). These iNGN cells were cultured and reseeded on MEAs adapting a previously described protocol (Schmieder et al., 2022). In brief, iNGN cells were thawed and cultured on Matrigel (Corning, Germany)-coated plates with mTeSR™1 medium (STEMCELL Technologies, Germany). Medium was prepared by mixing mTeSR™1 Basal Medium with mTeSR™1 5 × Supplement (STEMCELL Technologies, Germany) and adding 1% penicillin-streptomycin (P/S; Thermo Fisher Scientific, Germany). After passaging at least twice, cells were seeded in Matrigel-coated plates for induction. To initiate the induction process, 0.5 μg/ml doxycycline (Sigma-Aldrich, Germany) was added for three consecutive days. At 2 dpi Ara-C (5 μM final concentration; cytosine β-D-arabinofuranoside hydrochloride, Sigma-Aldrich, Germany) was added to suppress undifferentiated cells. Parallel to induction, MEAs were exposed to a plasma atmosphere (2 min, ambient air, 0.3 mbar, 50 W) and directly incubated with 0.1 mg/ml poly-D-lysine (PDL, Merck, Germany) solution overnight at 37°C. MEAs were then washed three times with sterile deionized water (10 min each) and dried under laminar flow. Laminin (0.05 mg/ml; Sigma-Aldrich, Germany) was then incubated overnight at 37°C. Induced cells were reseeded on the MEA chips (Multi Channel Systems, Germany) at 3 dpi. Cells were washed and then dissociated by incubating them with Accutase (Sigma-Aldrich, Germany) for 5 min. Cell suspension was centrifuged after taking cells up in their old medium at 359 g for 4 min. After re-suspending cells in complete BrainPhys™ medium, 100,000 cells in 1 ml media were given on one coated MEA. Complete BrainPhys™ medium was prepared by mixing BrainPhys™ Neuronal Medium (STEMCELL Technologies, Germany), 1% P/S, NeuroCult™ SM1 Neuronal Supplement (STEMCELL Technologies, Germany), N2 Supplement-A, 20 ng/ml recombinant human BDNF (Peprotech, Germany), 20 ng/ml recombinant human GDNF (Peprotech, Germany), and 200 nM ascorbic acid (Sigma-Aldrich, Germany). Since the iNGN culture after Ara-C treatment was of pure neuronal identity, astrocyte-conditioned media was added for enhancing long-term neuronal cultures. Half of the media was exchanged once a week with a 1:1 mix of fresh complete BrainPhys™ medium and astrocyte-conditioned complete BrainPhys™ medium.

Rat primary astrocytes (A1261301, Thermo Fisher Scientific, Germany) were cultured on coverslips coated with PDL and laminin according to the same procedure described above. After expanding the cells in astrocyte medium [DMEM with 4.5 g/l d-glucose, and pyruvate, N2 Supplement, 10% One Shot™ fetal bovine serum and 1% P/S (all provided by Thermo Fisher Scientific, Germany)], the medium was changed to complete BrainPhys™ medium after the culture being confluent. After culturing for at least 3 days, half of the medium was taken out as astrocyte-conditioned BrainPhys™ medium weekly and was replaced with fresh complete BrainPhys™ medium.

### 2.2. Experimental hardware

**FIGURE 1:**
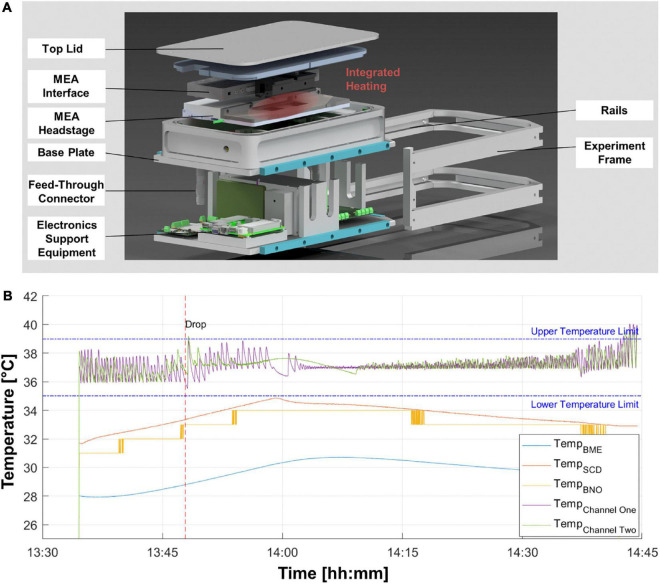
Environmental chamber for multi-electrode array (MEA) electrophysiology experiments on altered gravity platforms. **(A)** Exploded-view illustration of the experimental hardware depicting the main electrical and structural elements. **(B)** Temperature profile for one drop tower experiment session. The recording starts after the evacuation of the tower for a baseline recording of 15 min before the drop (red striped line). Purple and green lines depict the temperature at the two respective MEA chips. Other colored temperature lines are measured at different locations inside the pressure chamber.

The experiment module encases a MEA system (Multi Channel Systems, Germany) in a pressure chamber to keep the physiological conditions for the cell samples during the time of the experiment. Utilizing the integrated heaters of the MEA system, the cells are kept at their ideal temperature of 37°C. The pressure-tight encasing keeps constant pressure and CO_2_ level. A PC to record the electrophysiological data and store it redundantly on two physically separate hard drives is available outside the pressure chamber. The compact design makes the system inter-compatible with the most relevant altered gravity research platforms (DLR SAHC, ZARM drop tower, parabolic flights of airplanes and sounding rockets). For cell loading and unloading, a rail system was integrated to completely remove the experiment module. The easy access to the samples and the internal hardware improves the usability for time-sensitive operations, i.e., late access operations on sounding rockets. A scheme of the set-up with all main parts marked is shown in Figure 1A.

### 2.3. Centrifuge experiments

MEA cultures were transported to the SAHC at the DLR Cologne, Germany using a transport incubator (CellTrans 2018 and 4016, Labotect, Germany; 37°C, 5% CO_2_) 5 days before the first hypergravity exposure. Custom-made lids of poly(dimethylsiloxane) (PDMS) were used to cover the MEAs tightly (Blau et al., 2009). The PDMS lids kept the samples sterile, while allowing for gas exchange. The space between lid and MEA of all samples was filled completely with medium in an air-free way to avoid mechanical disturbances during transport and microgravity experiments. On the day of the drop, cultures were taken to the integration area of the drop tower in the transport incubator and integrated into the experiment chamber immediately. Temperature was kept at 37°C by heating plates and the chamber was flooded with synthetic air comprising 5% CO_2_ (Nippon Gases, Germany). Cells were placed in the experiment chamber mounted on the centrifuge on a swing-out platform, so that the resulting g-vector always acted perpendicular to the MEA chip surface avoiding inertial shear forces on the cells. The recording was started after remotely checking functionality of the system. After 10 min baseline recording, the g-level was elevated to 4 g or 6 g over a 30 s period (highest acceleration possible) and held for 5 min. After about 30 s of deceleration to 1 g, recording was continued for 5 min. Phase-contrast microscopy images were taken before and after each experiment run to confirm the viability of the neurons. Five MEA samples from one preparation were measured on three consecutive days (36–38 dpi), performing five runs with 4 g and six runs with 6 g.

### 2.4. Drop tower experiments

Neuronal cultures were transported to the drop tower facility in Bremen using transport incubators (CellTrans 2018 and 4016, Labotect, Germany; 37°C, 5% CO_2_) one week prior to the start of the experiments. The cells were handled as described previously for the hypergravity exposure to ensure comparability of the results (section “2.3. Centrifuge experiments”). After checking functionality of the system, the drop capsule, housing the experiment chamber as well as batteries and support electronics for data transfer, was pulled up and evacuation of the tower started. Exactly 15 min before the drop, all systems were started remotely, checked and recording was started. After 10 min of baseline recording the drop was initiated. Recording was continued until at least 10 min after the drop. Phase contrast microscopy images were taken before bringing the samples to the integration area and upon return to the cell culture lab. In total, five MEAs from three independent preparations were dropped on five consecutive days (46–55 dpi).

### 2.5. Data analysis

Raw data recorded by the 60 electrode chips (Multi Channel Systems, Germany) on a MEA2100 system (Multi Channel Systems, Germany) were transformed to binary files using the MC_DataTool (Multi Channel Systems, Germany) software. Since electrodes can record from several neurons, a spike sorting algorithm was employed to separate the activity of different units from the raw extracellular data. The SpikeInterface package (Buccino et al., 2020) was used for preprocessing the data and to run the Ironclust (Jun et al., 2020) spike sorting algorithm. The data was unitized using the whole recorded period from each drop or centrifuge experiment employing the software package for all further data handling on the whole data set. Preprocessing steps were included to denoise the data and to remove artifacts. Second order Butterworth high pass filtering (cutoff 100 Hz) and common median referencing was applied (Rolston et al., 2009). Spike sorting was then performed using default parameters except the detection threshold was set to 4.5. After spike sorting the output was automatically curated. All units with an interspike interval violation rate higher than 0.2 and a signal-to-noise ratio higher than five were accepted for further data analysis. The spike trains of all units passing this curation step were exported to NeuroExplorer 5 (Nex Technologies, USA). Mean firing and bursting rates were extracted. Bursting is a phenomenon seen in neurons and is characterized as a short bundle of spikes with a high internal frequency of spiking and longer periods of silence before and after. For burst detection an interval algorithm was used in NeuroExplorer 5. Following parameters were used for detection and have been described previously (Yang et al., 2018): 20 ms maximum inter-spike interval for starting the burst, 100 ms maximum inter-spike interval for ending the burst, 100 ms minimum inter-burst interval, 20 ms minimum duration of bursts, and minimum number of two spikes in each burst.

**FIGURE 2:**
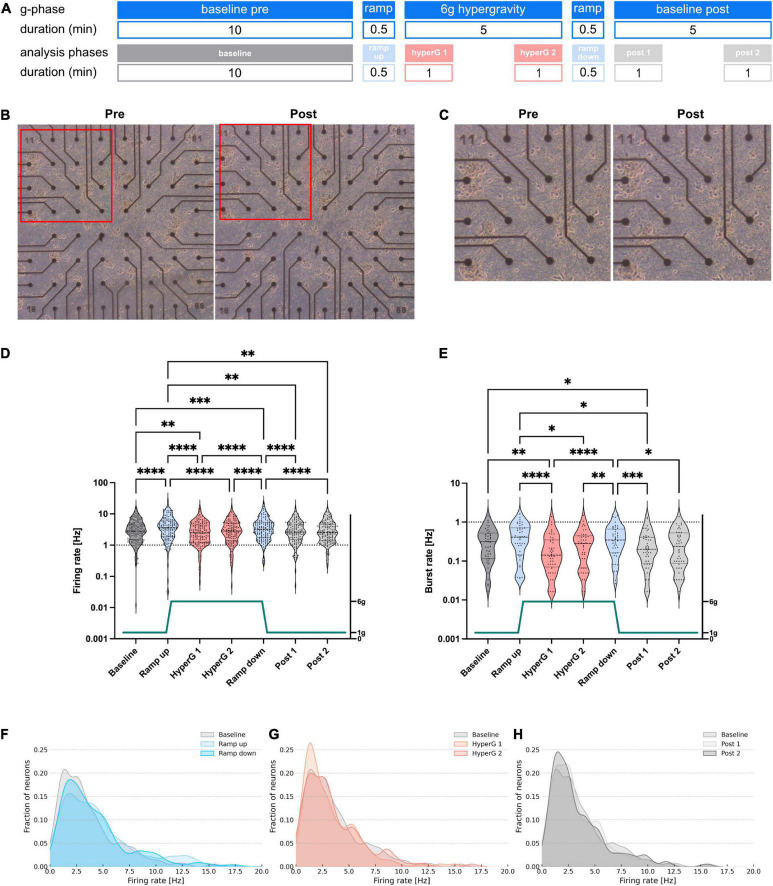
Investigation of morphology and electrophysiological activity of human neural networks subjected to 6 g hypergravity on a centrifuge. **(A)** Overview of the different experiment phases and the sections included in the analysis with their respective durations. **(B)** Phase contrast microscopy images of an exemplary MEA chip pre- and post-exposure to hypergravity. **(C)** Zoom images of the areas marked with a red frame in **(B)**. **(D)** Firing and **(E)** bursting rates of neuronal cultures subjected to 6 g hypergravity on a human centrifuge. Five MEAs were measured during six centrifuge runs. Statistical analysis included 144 and 44 units for firing and burst rate, respectively. Repeated measures one-way ANOVA and Tukey’s multiple comparisons test were used (*p < 0.05, **p < 0.01, ***p < 0.001, and ****p < 0.0001). Dashed lines mark the first, second, and third quartile in the violin. **(F–H)** Density plots of the firing rates during ramp **(F)**, hypergravity **(G)**, and post-exposure **(H)** phases compared to the baseline.

**FIGURE 3:**
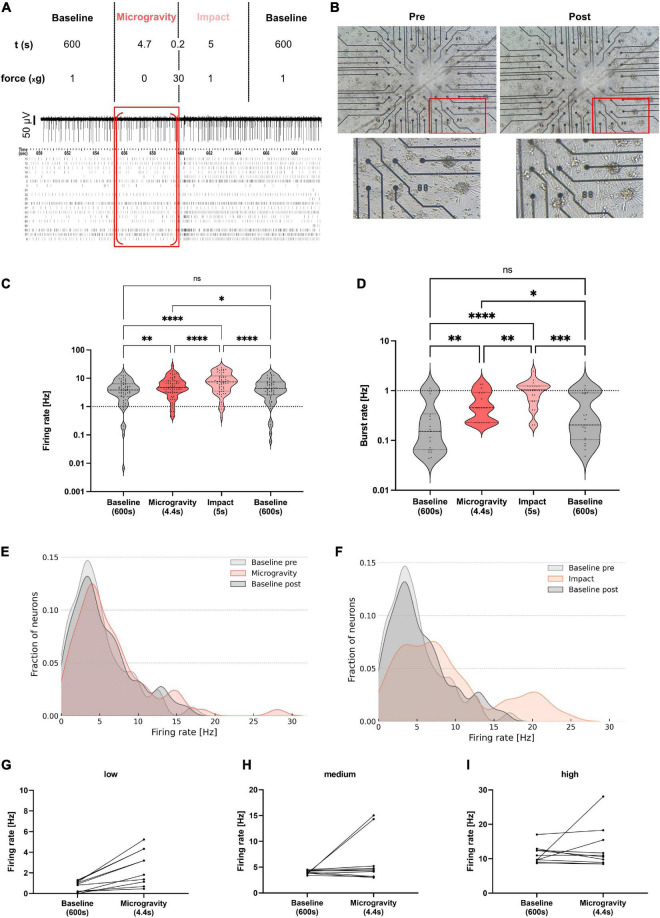
Electrophysiological activity of human neural networks subjected to microgravity in a drop tower experiment. **(A)** Overview of the different experiment phases with respective g-levels and durations. A representative electrode trace and a raster plot of all neurons of one exemplary MEA chip is shown with the microgravity phase marked with a red frame. Red brackets mark the section excluding safety margins which was used for analysis. **(B)** Phase-contrast microscopy image of an exemplary MEA chip pre- and post-drop. A zoomed area (red frame) of the culture is shown below. **(C)** Violin plot for comparison of firing rates averaged during baseline recordings prior to the drop (grey, left), during the microgravity phase (orange), during the impact (light orange), and post-drop baseline (grey, right). Five MEAs from three independent preparations were measured during five drops. Statistical analysis included 63 and 19 units for firing and burst rate, respectively. Repeated measures one-way ANOVA and Tukey’s multiple comparisons test were used (ns, not significant; *p < 0.05, **p < 0.01, ***p < 0.001, and ****p < 0.0001). Firing rates of individual units are marked with a black triangle in each condition. Dashed lines mark the first, second, and third quartile. **(D)** Violin plot for comparison of burst rates during all four phases. **(E,F)** Density plots of the firing rates during microgravity and impact phases compared to baseline pre- and post-drop. **(G–I)** Subgroups of neurons show a distinct change in firing rate between baseline and microgravity. Units with low **(G)**, medium **(H)**, and high **(I)** firing rates during baseline and their respective firing rate during microgravity (dots connected by a line).

For the centrifuge experiments, recordings were split into seven different phases for analysis: Baseline (10 min), Ramp up (∼30 s), Hypergravity 1 (1 min, hyperG 1), Hypergravity 2 (1 min, hyperG 2), Ramp down (∼30 s), Baseline post 1 (1 min), Baseline post 2 (1 min). For the hypergravity phase (5 min total) and the following baseline (Baseline post, 5 min total), the first and last minute were analyzed separately to check for potential adaption processes. An overview of the centrifuge experiment and analysis phases is given in Figure 2A. For centrifuge recordings, we applied safety margins of 2 s at the beginning and end of all phases before extracting firing and bursting rates. This was an additional security measure to make sure that all phases were cleanly separated and no overlap was falsely attributed to another phase. For the drop tower experiments, values were extracted for five drops during four phases: Baseline (600 s), Microgravity (4.7 s), Impact (5 s), and Baseline (600 s). Safety margins of 150 ms were deducted at the beginning and end of all phases. An overview of the drop experiment and analysis phases is given in Figure 3A. Since firing and bursting rates are lognormally distributed, the logarithm of the values was taken for statistical analysis (Buzsáki and Mizuseki, 2014). All units that did not show any activity in one of the phases were completely removed from the analysis to avoid inclusion of artifacts. Statistical analysis of the centrifuge experiments included 144 and 44 units for firing and burst rates, respectively. In the drop tower experiments 63 and 19 units were included for firing and burst rates, respectively. Prism 9 (GraphPad, USA) was used for statistical analysis and visualization. A repeated measures one-way ANOVA with the Geisser–Greenhouse correction and Tukey’s multiple comparisons test was used for statistical analysis and comparison of the experiment conditions. Density plots of the firing rate distribution were created using a custom Python script.

For the analysis of changes in AP waveform during the different experimental phases, we extracted AP amplitude and half width of the waveform templates. Parameters were exported for all units detected after spike sorting and automatic curation as provided by SpikeInterface and Ironclust sorting algorithm. Values were imported into Prism 9 and outliers were detected using the ROUT method with Q = 1%. Remaining values were analyzed using a non-parametric ANOVA (Friedman’s test).

## 3. Results

### 3.1. Environmental chamber provides stable experiment conditions

Biological experiments under altered gravity conditions are particularly difficult to implement. Creating hyper- or microgravity conditions is not easily achieved in a standard laboratory setting and thus demands the samples to be placed in new environments by means of specialized hardware. Moreover, access to gravity research platforms is scarce and costly, as mostly vast efforts need to be performed to achieve high-quality hyper- and microgravity conditions on Earth. Thus, any device needs to be modified and custom-designed to conform with the desired gravity research platform. Accordingly, we developed a reusable module, enclosing the entire MEA system including the headstage and interface board (Figure 1A). This experiment hardware provides and maintains a constant air composition, pressure, and temperature with the possibility to monitor these parameters. The experiment module records the environmental conditions inside the pressure chamber. Exemplary data for the first drop of the campaign at ZARM drop tower is shown (Figure 1B). Position sensors provide the acceleration of the experiment module and show the maximum g-loading in all directions. The acceleration data of the module was compared and verified with the data from the inertial measurement unit of the drop capsule. The maximum deceleration of 38.2 g was measured in the first drop.

Neurons are sensitive to temperature fluctuations, which lead to the requirement of an active temperature control for each MEA chip. Temperature graphs in Figure 1B show optimal conditions, as the temperature stays within the boundaries of temperature deviation of ±2°C depicted for various temperature sensors located within critical points in the experiment module. A small overshoot is acceptable as the inertia of the heat plate, the glass chip and the medium of the cells provides a dampening of temperature fluctuations to keep the temperature of the cells within the desired limits. The pressure, humidity and CO_2_ levels were within the optimal ranges. The humidity levels were kept below 35% RH creating an optimal condition for the electronics inside the pressure chamber. The CO_2_ level remained above 4% and the pressure inside the experiment chamber was successfully kept within the limit of ±40 mbar of atmospheric pressure during the entire experiment.

Since the MEA system records electrophysiological signals with a high frequency (25 kHz) and fidelity (μV range), the experiment data had to be controlled for electromagnetic noise and other interference that could lead to false-positive phantom spikes. On the centrifuge as well as the drop tower, the payload quickly moves not only through the Earth magnetic field, but also through many wireless signals, metal structures and running electrical devices, which could lead to interference and noise. The environmental chamber includes few moving mechanical parts, but vibration and changes in the gravitational load on the structure could also lead to noise. To confirm the integrity of the recorded data, various tests and controls were performed. The system was tested on the centrifuge with empty MEA chips and accelerations of up to 6 g, which did not lead to any increase in background noise in the single MEA channels. Because in every experiment in the drop tower or on the centrifuge, two MEA chips were recorded simultaneously, any systemic interference should appear in both chips at the same time, so potential phantom spikes or other interference can be cross-checked in both recordings. In addition, the neural network does not cover the whole recording area. Therefore, in each MEA chip some channels do not show any captured activity. These empty channels with no electrophysiological data were used to control for interference or noise-induced spikes by running the data through the spike detection algorithm. No spikes were detected in the empty channels on the centrifuge in hypergravity or in the microgravity or impact phase of the drop tower experiments. This confirms that the payload is resistant to any induced interference, electromagnetic noise, or phantom signals evoked by increased or reduced mechanical loading. Additionally, safety margins were included in the data analysis that only clean data without overlap into other gravity phases or noise were included. Thereby, signals falsely detected as spikes were excluded from our data.

### 3.2. Centrifugal-induced gravitational stimuli evoke functional responses in neural networks

To investigate the influence of hypergravity exposure on the activity of hiPSC-derived neural networks, the experimental hardware was installed on the SAHC (DLR, Cologne, Germany). After insertion of two chips into the MEA system within the experiment module, a 10 min baseline recording of spontaneous non-evoked electrophysiological membrane potentials was performed as the 1 g normal gravity control, before exposure to 6 g hypergravity for 5 min, followed by another 5 min baseline recording after centrifugation to assess potential adaptation processes (Figure 2A). To control for standard internal fluctuations of neural activity we measured MEAs over the same period without the application of a gravity stimulus. We did not find any significant differences in measured firing rates between phases without applying gravity changes (Supplementary Figure 1). Phase contrast microscopy before and after the experiment was performed to verify neural network viability and integrity. No adverse effects on the cells were detected after the experiment runs by assessment of morphological appearance (Figures 2B, C).

For firing rate analysis, data sets recorded during the different phases of the experiment were addressed separately. In order to detect any potential processes of adaptation, for the hypergravity and the baseline post phases, two one-minute data samples were analyzed: one at the beginning and one at the end of each phase, respectively (Figure 2A). When the hypergravity level of 6 g was reached (hyperG 1), a decline in firing rates was observed (Figure 2D). However, the late-phase hypergravity data (hyperG 2) did not show any significant variation from the baseline recording, hinting at an adaptation mechanism to occur. Upon going back to 1 g conditions (ramp down) once more, a rise in activity was observed. When reaching a constant level of 1 g again, firing rates went back to baseline values within a time frame of 1 min. Furthermore, no significant alterations in activity were observed between beginning and end of the re-adaptation phase after hypergravity exposure (data samples post 1 and post 2).

The dynamics of activity changes became more visible for the mean burst rate profile. Here, there was a noticeable rise in activity during ramp phases, a subsequent activity dip during start of the 6 g hypergravity period, followed by a convergence to baseline levels after prolonged periods of 5 min constant exposure to 6 g hypergravity (Figure 2E). The re-adaptation of burst rates following deceleration did not occur within the first minute but within a time frame of 5 min, which indicates active re-adaptation processes, as passive adaptation would occur instantaneously. However, changes between baseline and ramp data sets of burst rates were not significantly different as compared to firing rates.

Data recorded in a similar experiment with hypergravity exposure to 4 g yielded no significant changes in firing rates between all phases except for the deceleration (ramp down) phase and the second analyzed period following exposure (post 2). Burst rate differences were only significant considering the baseline control compared to the acceleration (ramp up), deceleration (ramp down) and 5 min re-adaptation phase (post 2) (Supplementary Figure 2).

Here, the importance of mechanical stimuli with respect to neuronal activity is emphasized, as mostly acceleration and deceleration but not constant exposure to hypergravity yielded significant changes in spontaneous neuronal activity. To illustrate how firing rate values of single measured electrode units were distributed and potentially shifted during the different experiment phases, a density plot was used (Figures 2F–H). A shift of firing rates could be measured toward higher frequencies during the ramp phases. Local maxima emerged between 8 and 13 Hz for both, acceleration and deceleration. During acceleration, frequency distribution shifted more than during deceleration (Figure 2F). During the hypergravity phase, a much larger fraction of units was allocated in the lower firing rate range (around 2 Hz), which was observed particularly in the hypergravity phase immediately following the ramp up (hyperG 1, Figure 2G). Nonetheless, units with high firing rates persisted as well. This suggests that different subgroups of measured neurons exist that were not affected comparably by the gravity stimuli. However, these units seemed to be able to recover, as the shape of the second analyzed hypergravity (hyperG 2) phase resembled the baseline. Comparing baseline and post-exposure phase distributions, only slight alterations are visible (Figure 2H). The fact that the shape of the distribution as observed during baseline is not simply shifting to higher or lower firing rates in other experiment phases is indicating that not all neurons react in the same way to changes in gravity. Thus, the analysis of different neuronal subgroups might be necessary in future experiments. Our data also suggests a change in the shape of the APs. Amplitude and half width of the spikes changed significantly as a response to gravity (Supplementary Figure 3).

### 3.3. Electrophysiological activity of neural networks is elevated during a short-term microgravity phase

The hypergravity experiments suggest that neurons react to gravitational stimuli with changes in mean firing and burst rates as well as different timelines for re-adaptation. Responses in neural firing rate were observed during the ramp up and down phases and throughout the 6 g hypergravity phase. As neuronal activity now seemed to be regulated by gravity-sensitive mechanisms, exposure to microgravity would likely evoke a response as well. To investigate the impact of mechanical unloading by microgravity conditions, the MEA experiment module was integrated on a drop tower platform, enabling to subject the networks to a high-quality level of microgravity (10^–6^–10^–5^ g) for 4.7 s.

To check for integrity and morphological changes in the neural networks, microscopy images of all samples were taken before and after the drop. An exemplary culture is shown in Figure 3B. For all five samples, the networks were stably attached to the MEA after the drop. No visible morphological changes could be observed.

Electrophysiological activity of the networks was measured, starting with 1 g baseline recording as a control for 10 min before each drop (Figure 3A). The recording was continued throughout the microgravity phase obtained while subjecting the cells to microgravity in free fall, followed by the impact with a resulting hypergravity impulse of around 30–40 g. After the impact, the recording was continued for another 10 min to measure the re-adaptation phase after the exposure (post-drop baseline). Splitting the recorded data according to these baseline (normal gravity control), microgravity as well as impact, and post-exposure baseline (re-adaptation) phases resulted in four data sets (Figure 3A). Data of the same phase from all drops were combined and analyzed regarding the neural network firing and burst rates.

Comparing the firing rates of the human neural networks on the MEA chips showed a significant increase from the baseline to the microgravity phase, suggesting an increased activity of the neural network under microgravity conditions (Figure 3C). From the microgravity phase to the impact phase, a further significant increase in neuronal firing rate was measured. Over the time of the post-drop baseline, the firing rate was significantly lower again than during the micro- and hypergravity phases, with no significant difference to the pre-drop baseline, indicating that cells recover from altered gravity influence. The firing rate decreased back to baseline activity within 10–20 s (Supplementary Figure 4).

The burst rate analysis of the drop tower MEA data revealed similar dynamics as the analysis of the firing rate. Comparing the baseline and the microgravity phase, a significant increase in neural network burst rate was measured. Another rise in activity comparing the microgravity phase to the hypergravity impulse of the impact could be measured (Figure 3D). After the impact, the burst rate normalized again within 10 min to baseline levels. Since the 1 g baseline control measurements were very similar before (pre-drop) and after the drop (post-drop), the robustness as well as re-adaptation capability of the neural network could be depicted.

Distribution of the single unit firing rate values was again illustrated in a density plot. The shape of the curve for pre- and post- baseline profiles was similar. The baseline peak firing rate of around 3 Hz shifted to 4 Hz in the microgravity phase and more data points were collected at the higher frequency part of the spectrum (15–28 Hz) (Figure 3E). Concurring with the observations made in the statistical analysis of firing rates, the shift toward higher firing rates was even more visible for the impact phase data. Here, firing rate values showed an altered activity frequency distribution with two major peaks at 8 and 20 Hz (Figure 3F). To see in more detail how subpopulations of neurons react to microgravity the firing rate of different units was assessed for changes from baseline to microgravity levels. The firing rates of the 10 lowest and highest firing units as well as the 10 units in the middle of the spectrum at baseline were plotted. The change of firing rate from baseline to microgravity phases was also marked for each unit (Figures 3G–I). All low firing units increased their firing rate from baseline to microgravity (Figure 3G). Few medium firing units also decrease their rates with most showing an increase (Figure 3H). High firing units reacted in a mixed way with about half of the units increasing and half decreasing their firing rates (Figure 3I). Looking at the results presented in the density plots and changes of individual units of the low, medium, and high firing regime it can be concluded that not all units react in the same way to changes in gravity. We observe a complex pattern of responses to gravity change when moving from the population to the single unit level.

These electrophysiological measurements show that neural networks react to changes in their gravity environment with adaptations of spontaneous activity. Moreover, AP amplitude decreased significantly comparing baseline to impact phase (*p* < 0.05, Supplementary Figure 3), while no significant change of AP amplitude could be measured for the other phases. AP half widths also did not change between phases (Supplementary Figure 3). Acute gravity changes resulted in most pronounced and immediate effects. Microgravity exposure led to significant increases in firing and burst rates over the course of less than 5 s. Furthermore, the functional viability of the cells was not influenced by the microgravity phase or the impact, confirmed by the successful re-adaptation of neuronal activity back to baseline values as well as assessment of the cells’ morphological appearance prior to and following the drop experiments.

## 4. Discussion

Investigations in the field of gravitational biology can often only be answered employing large, complex, and expensive research platforms. Access to these advanced facilities is scarce and the biological samples as well as the experiment hardware need to endure extreme environmental stimuli, e.g., in the form of high mechanical forces. Astronauts that are subjected to microgravity conditions often suffer from neurological phenotypes. Studies on neuronal systems at a cellular level are emerging but only a few experiments have been conducted until now (Meissner and Hanke, 2002, 2005; Pani et al., 2013; Sieber et al., 2014; Genchi et al., 2015; Hauslage et al., 2016; Kohn et al., 2017; Cui et al., 2018; Kohn and Hauslage, 2019). To understand the impact of gravitational loading conditions on brain function, further studies are necessary that investigate crucial mechanisms, such as neuronal transmission rates. Neuronal activity can be directly related to structural changes in neuronal morphology as well as mechanistic biochemical changes in pathways underlying synaptic transmission. Thus, electrophysiological assessment of neuronal activity employing a MEA system was chosen as a highly sensitive measurement technique that generates reproducible and robust data sets in the millisecond range even under extreme environments, such as altered gravity conditions.

With our study we demonstrate the feasibility of electrophysiological measurements using human neural networks with a MEA system under altered gravity conditions, which are challenging for both the experiment hardware as well as the applied neurons. Our experimental set-up consisted of a pressure chamber, various sensors monitoring environmental conditions, the MEA measurement system, and a heating unit, to maintain optimal experimental conditions for reliable results (Figure 1). The MEA experiment module was able to maintain viable physiological conditions for the neurons over extended periods of time (at least 3 h). Outside the pressure chamber the support electronics (i.e., a temperature controller, computer for acquiring, and redundantly storing the data from the MEA system) enabled remote or autonomous operation of the experiment. Our set-up was designed to be adaptable to perform future experiments on other experimental platforms, such as in sounding rockets or parabolic flights of airplanes. Furthermore, the set-up could be used to investigate other electrically active cell types such as cardiomyocytes or acute brain slices or brain organoids. Integration of a high-density MEA system in the set-up would allow for even more detailed investigation of network activity.

During the centrifuge and drop tower campaigns we could show that our set-up is providing constant conditions with respect to temperature, pressure and CO_2_ levels, all necessary for cell experiments. In both campaigns no influence on cell morphology was observed, which is indicative that cells remained fully viable. The module is capable to withstand the conditions of altered gravity platforms such as high g-forces.

The implementation of the experiment hardware on the SAHC confirmed suitability of the novel MEA module for real-time measurement of electrophysiological activity also under hypergravity conditions (Figure 2). Our data revealed that neurons react to gravitational stimuli very quickly with significant changes in firing and burst rates. These reactions, however, are reversible as no differences between baseline recordings prior to and after the exposure to altered gravity could be detected. Important to note is that the neurons responded differently to each gravitational loading condition. Acceleration and deceleration during ramp phases on the centrifuge, induced increased activity. Opposed to the acute acceleration phases during the ramps, constant hypergravity phase with a gravitational force of 6 g induced decreased activity (Figure 2). The rise in firing rates during the ramp phases could be explained by the accompanying mechanical forces on the system and the well-established presumption that neurons are mechanosensitive. Indeed, it has been demonstrated that activation of mechanosensitive ion channels can trigger spiking in hippocampal pyramidal neurons (Nikolaev et al., 2015) and the role of mechanosensitive receptors in neuronal excitability has been discussed for years (Kirischuk, 2009). Earlier experiments using calcium imaging in SH-SY5Y cells on a parabolic flight did show an increase in fluorescence under hypergravity, and a slight decrease during microgravity (Kohn, 2013). This could be explained by mechanosensitivity of Ca^2+^-Channels. Furthermore, mechanosensitive ion channels and gated ion transport are in the focus of being key elements in the perception of gravity (Häder et al., 2017).

The fact that neuronal network activity declines below baseline levels upon reaching a constant level of 6 g hypergravity indicate another cellular mechanism. One explanation is that the cells overcompensate for the raise in activity they experience during the preceding ramp phase. Neurotransmitter receptor attenuation by prior overexcitation might induce a rapid adaptation after acceleration phases. This adaptation mechanism to keep up a constant level of signaling would also explain why the activity did not diverge from the late hypergravity phase to baseline measurements. Accordingly, late-phase hypergravity and post-experiment data similarly do not differ intrinsically. These results lead to speculate that acute changes in gravity levels, i.e., the acceleration and deceleration forces, most prominently induce activity changes in neural networks. Constant increase in mechanical load by elevated g-forces induced less pronounced but very significant opposing effects of attenuated neuronal activity. This decreased activity pattern at 6 g is reversed after 5 min of constant exposure to hypergravity, at which time point activity levels of both firing and burst rates are similar to the control conditions at 1 g. Interestingly, after deceleration the firing rate readapted rapidly within 1 min to baseline levels, while the burst rate was still significantly decreased and an adaptation back to baseline levels occurred only during the course of 5 min.

The firing frequencies changed in a gravity-dependent way most likely within specific neuronal subgroups that still need to be identified. The most pronounced effect was an increase in firing rates of low-activity (2 Hz) neurons, but also medium-active neurons in the range of 6 and 9 Hz increased their activity.

Another hypothesis is that hypergravity indeed lowers firing rates due to changes in the membrane fluidity (Kohn et al., 2017) but that cells are capable to adapt and overcome this interference within a short period of time (few minutes). Measurements carried out under 4 g conditions did not yield significant alterations in firing and burst rates, although the trend in firing rate development was similar (Supplementary Figure 2). This suggests that there might be a certain threshold in level of hypergravity at which effects become more pronounced or that acceleration forces up to a 4 g level were not high enough to induce significant effects.

AP amplitude and AP half width showed significant changes during hypergravity compared to baseline before and after altered gravity, which might be linked to altered ion channel dynamics due to changes in membrane fluidity (Kohn and Ritzmann, 2018). Although, the biological relevance of a change in mean AP amplitude from 30.52 to 30.76 μV has to be questioned. Baseline to baseline comparison showed also significant differences, which indicates that re-adaptation processes need a longer time period (Supplementary Figure 3). Therefore, in future experiments the data collection will be adjusted to identify potential re-adaption processes.

Further studies are needed to investigate the underlying mechanisms of gravity-dependent changes in neuronal transmission especially within different sub-populations of neurons within the network. Experiments employing longer phases of hyper- and microgravity are necessary to investigate the hypothesis of adaptive processes following acute acceleration and thus mechanical stress, e.g., by sounding rocket experiments. Furthermore, studies with repeated acceleration and deceleration phases, e.g., in parabolic flights, could be conducted to investigate whether the activity-enhancing effect of acute acceleration phases can be replicated consistently. Here, in regard to the observed elevated firing rates during the first ramp phase, it will be interesting to see if neurons become more resistant to the altering gravity cue with time or if acceleration force boosts cell reaction in an additive way compared to deceleration.

The observation that neural networks can sense and react to gravity changes was further confirmed by the drop tower experiment, where we noticed a significant increase of firing rates during the 4.7 s microgravity phase as compared to baseline measurements (Figure 3). Microgravity seemed to induce opposing effects to constant hypergravity, as neuronal activity was increased during micro- and decreased during hypergravity phases. Here, both firing and burst rates showed similar results of enhanced activity during microgravity exposure.

As expected, from our hypergravity studies, the impact of the drop capsule of approximately 30 g for few milliseconds following the microgravity phase had a large effect neuronal activity. This is in line with our centrifuge experiments, where acute acceleration from lower to higher gravity levels (ramp phases) lead to elevated firing rates, likely due to mechanosensitive mechanisms of the neurons. This raises the question whether the same process is occurring *in vivo* during traumatic brain injury. If so, exposing neural networks to short but high g-levels could serve as a model for changes in brain function as a result to such injury. Concurrent with the centrifuge experiments, cells recovered very quickly from the micro- and hypergravity phases of the drop, as firing rates recorded 10 min after altered gravity conditions did not significantly differ from 1 g baseline firing rates. The system in the drop tower releasing the experiment module within the drop capsule causing neglectable mechanical stimulation, allows for immediate progression from 1 g normal gravity to high-quality microgravity during the 4.7 s of free fall. An overcompensation that would support our previous hypothesis could not be observed in the analysis phases chosen here. Neural network activity changes detected during the microgravity phase raise the question whether the increase in firing rates was again due to mechanical stimuli caused by the change to force-free conditions during free fall and perceived by the cell or due to another cellular mechanism as a direct reaction to microgravity. During microgravity especially low-activity neurons increased their frequency of APs from approximately 3–4 Hz. The high-activity neuron sub-population increased firing at ranges of 15–28 Hz as well. In contrast to the microgravity phase, the mechanical strain derived from the impact induced an increase in neuronal activity around 8 and 20 Hz of firing frequencies. The finding that sub-populations are reacting differently to the change from baseline to microgravity is supported by looking at individual neurons. Low firing units increased their firing rate whereas high firing units reacted with about 50% increasing and 50% decreasing their firing rates. Whether these differences come from single neurons reacting differently to gravity or from the whole network dynamics and thus single neuron firing being altered as a result of the gravity, still needs to be clarified.

Future experiments are necessary to assess the responsible mechanisms and neuronal sub-populations undergoing the differential changes in activity patterns. Acute mechanical stimulation by acceleration forces should be assessed in more detail. Comparisons to hyper- and microgravity phases might need prolonged phases of altered gravity exposure especially for microgravity experiments. Adaptation of neuronal activity is regulated differentially as firing and burst rate re-adaptation time frames differ after hypergravity and after acceleration phases. To investigate adaptive processes in more detail, other gravity research platforms need to be taken into consideration, such as the GraviTower Bremen Pro allowing for repeated exposures with variable accelerations, parabolic flights with alternating hyper- and microgravity phases (ca. 22 s) or sounding rocket campaigns for intermediate-duration exposure (5–6 min) to microgravity. Follow-up experiments should also investigate the reaction of different neuronal cell types to gravitational stimuli, since biophysical differences could affect their response profile. Additionally, the AP amplitude and AP half width should also be closely monitored with regards to potential changes mediated by altered gravity. Responses to altered gravity could potentially be affected by maturation state of the same network, which is why different culture ages should also be studied as well. To understand the molecular mechanisms behind our observation, further studies with ion channel knock-out models are required. Alternatively blocking or inhibiting certain molecular targets of interest could help in mechanistic studies. Methods like transcriptomic analysis of the samples would support these efforts. Other studies could even move from *in vitro* to *ex vivo* experiments using brain slice cultures.

Long-duration adaptation can only be conducted in orbital space vehicles (satellites and space stations), where opportunities for research applications are even less accessible. Thus, exposing neural networks to hypergravity and microgravity conditions should be conducted in ground-based studies on Earth as well. Hypergravity can easily be achieved by centrifugation, e.g., employing the MuSIC incubator-centrifuge at DLR (Frett et al., 2016; Lichterfeld et al., 2022). Microgravity can be simulated on Earth most reliably by counteracting Earth’s gravity vector using a 2D fast-rotating clinostat (Hemmersbach et al., 2006). Additionally, further assays should be initiated to decipher whether altered gravity-evoked modifications in membrane fluidity could be an explanation for the observations (Sieber et al., 2014; Kohn et al., 2017).

Understanding the different receptor potentials and neuronal subgroups will need applications of pharmacological interventions that e.g., block certain receptors specifically. Until now, spontaneous activity changes were investigated, but evoked potentials and responses to acute membrane potential stimuli during exposure to altered gravity were not assessed so far. The identification of potential pathways or target molecules for the development of countermeasures against detrimental effects of space conditions should be conducted in the future. Countermeasures against detrimental effects of altered gravity will be beneficial not only for manned space exploration missions, but also for patients on Earth that suffer from neurological disorders with similar disturbances of neuronal transmission efficiencies.

In summary, we could show that the utilized cell model is suitable to investigate neuronal activity changes already for very short phases of altered gravity of few seconds, as the cells reacted immediately to altered environmental conditions and mechanical loads. Additionally, activity measurement by MEA technology has proven to be a suitable readout technique sustaining even extreme environmental conditions and mechanical strain on the experiment hardware. Our results emphasize that more research is needed on how altered gravity conditions affect the electrophysiological processes within our brain.

## 5. Conclusion

With our study we have collected a large data set of electrophysiological activity of networks of human iPSC-derived neurons under altered gravity conditions. To achieve this, we constructed and built an experiment module facilitating the cultivation of neurons and simultaneously measuring their activity patterns in millisecond resolution in various experimental environments. We could confirm the functionality and integrity of the module in different conditions, namely the drop tower in Bremen, Germany, and the SAHC at DLR in Cologne, Germany. This is the basis for future and more advanced experiments of such kind in extreme conditions or space-related research platforms. Our results confirmed the immediate impact of altered gravity conditions on the electrophysiological activity of human neurons. Taken together, microgravity caused an increase in activity whereas hypergravity attenuated neuronal activity. This is in line with the sparse previous results of other cellular model systems. Further research will be needed to investigate the response of neural networks to altered gravity conditions in more detail. Our results suggest that sub-populations of neurons respond differently to the change in gravity or that the network as a whole is adapting in a more complex way. To get more detailed insight into this, experiments with extended research opportunities for altered gravity applications on various experiment platforms will be necessary.

## Data availability statement

The raw data supporting the conclusions of this article will be made available by the authors, without undue reservation.

## Author contributions

JS, LK, MS, ND, SP, YL, and CL: conceptualization and methodology. JS, MS, and RH: software and validation. JS, LK, MS, ND, SP, and YL: formal analysis, investigation, visualization, data curation, and writing—original draft. JS, LK, MS, ND, SP, YL, RH, JH, VB, SES, and CL: writing—review and editing. JS, LK, MS, ND, SP, YL, VB, SES, and CL: supervision, project administration, resources, and funding acquisition. All authors contributed to the article and approved the submitted version.
