# Supplementary material for: Human neural network activity reacts to gravity changes in vitro
Source: Front Neurosci. 2023 Mar 8;17:1085282. doi: 10.3389/fnins.2023.1085282 (PMC10030604; doi:10.3389/fnins.2023.1085282)
Supplement: Supplementary file 1 [file Data_Sheet_1.pdf]

Supplementary Material

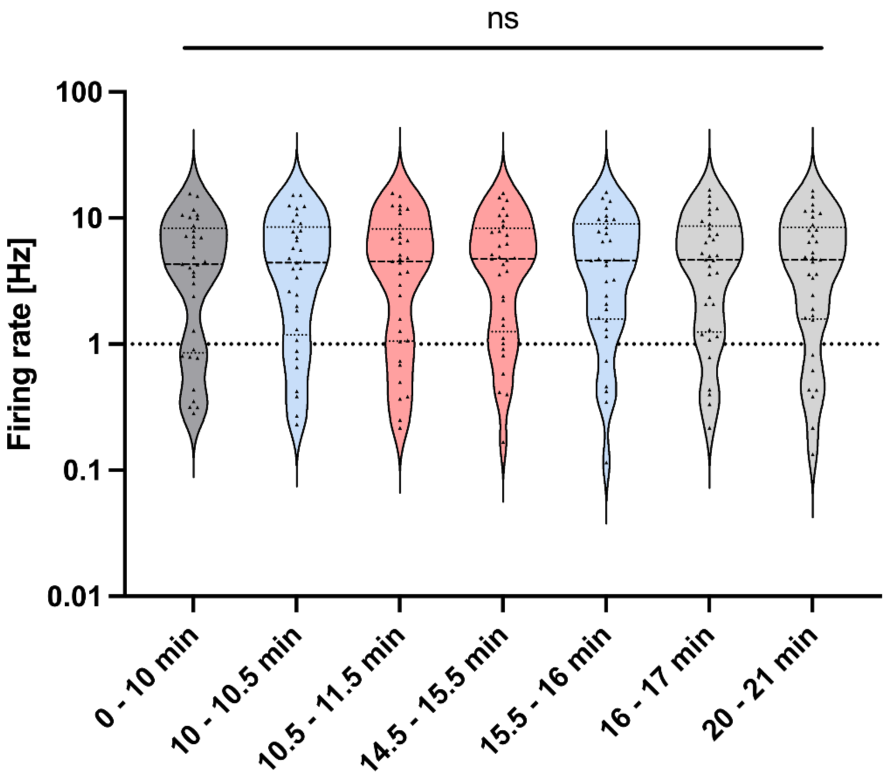

Supplementary Figure 1. Electrophysiological activity of human neuronal networks during different time points under constant 1 *g*. This was to control for fluctuations of activity in longterm MEA recordings. Violin plots of firing rates averaged over seven time phases of the recording (same duration and timing of data as in centrifuge experiment): first 10 min, 10 - 10.5 min and 15.5 - 16 min (blue), 10.5 - 11.5 min and 14.5 - 15.5 min (red), 16 - 17 min and 20 - 21 min (light grey). 37 units of 5 MEAs (25-94 dpi) from 5 independent preparations were included. Firing rates of individual units are marked with a black triangle in each condition. Dashed lines mark the first, second and third quartile (ns not significant).

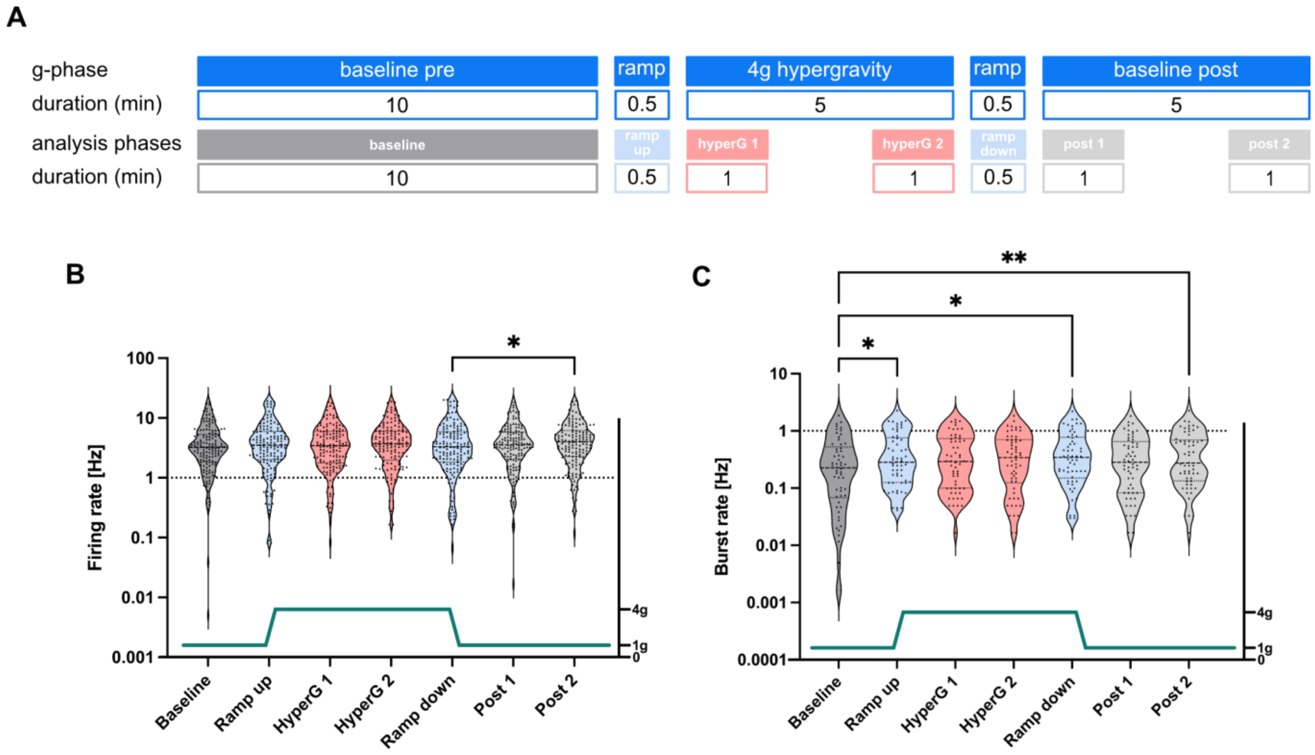

Supplementary Figure 2. Investigation of electrophysiological activity of human neural networks subjected to 4 *g* hypergravity on a centrifuge. (A) Overview of the different experiment phases and the sections included in the analysis with their respective durations. (B) Firing and (C) bursting rates of neuronal cultures subjected to 4 g hypergravity on the centrifuge. Five MEAs were measured during 5 centrifuge runs. Statistical analysis included 153 and 58 units for firing and burst rate respectively. Dashed lines mark the first, second and third quartile in the violin. (* $p<0.05$ and ** $p<0.01$)

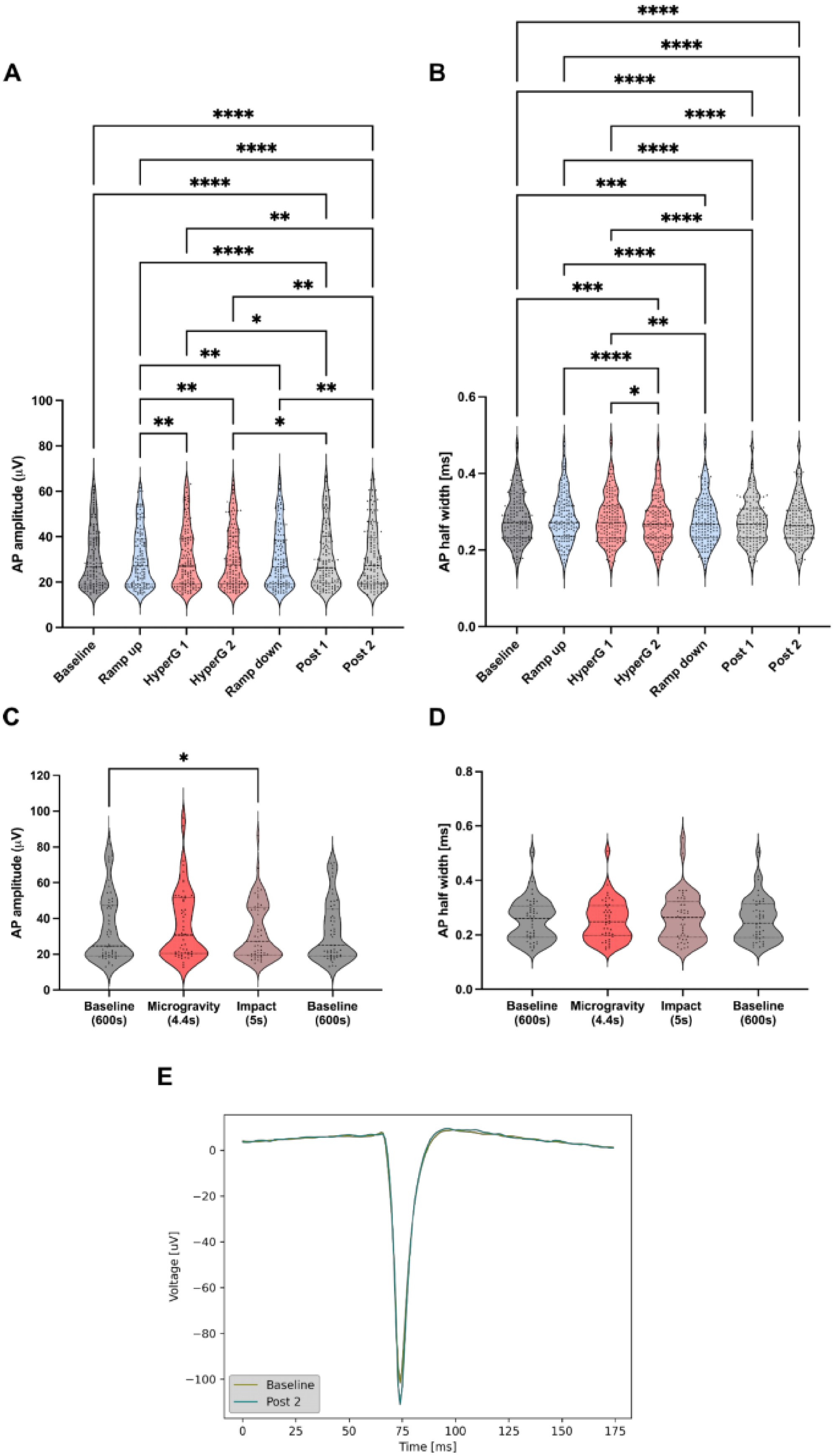
A
B
C
D
E
AP amplitude (µV)
AP half width [ms]
Baseline
Ramp up
HyperG 1
HyperG 2
Ramp down
Post 1
Post 2
Baseline (600s)
Microgravity (4.4s)
Impact (5s)
Voltage [uV]
Time [ms]
Baseline
Post 2

Supplementary Figure 3. Analysis of the AP waveform during the centrifuge and drop tower experiments. Templates of the waveforms as detected by the spike sorting algorithm were analyzed during the different experimental phases. (A) AP amplitude and (B) AP half width of neuronal cultures subjected to 6 *g* hypergravity on the centrifuge. (C) AP amplitude and (D) AP half width of neuronal cultures subjected to microgravity in the drop tower. (E) Exemplary waveform template of the same unit during Baseline and Post 2. Statistical analysis: Amplitude and half width of templates were exported as provided by Spikeinterface and Ironclust sorting algorithm. Values were imported into Prism 9 (GraphPad) and outliers were detected using the ROUT method with Q=1 %. Remaining values were analyzed using a non-parametric ANOVA (Friedman's test). (ns not significant, * p<0.05, ** p<0.01, *** p<0.001, and **** p<0.0001)

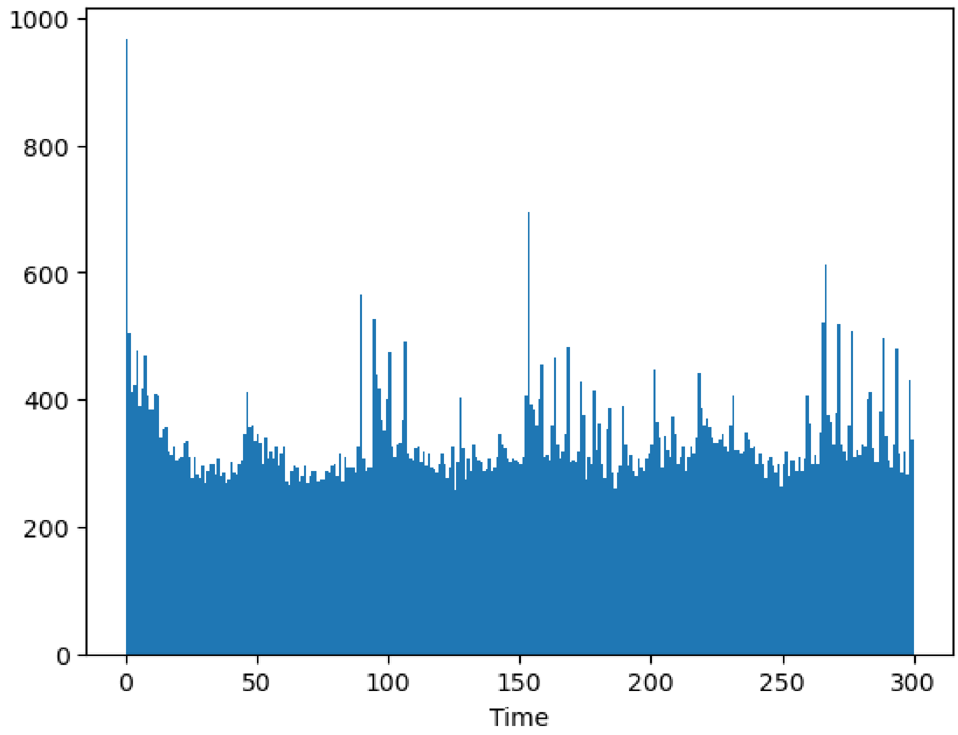

Supplementary Figure 4. Histogram of cumulative spikes over time after impact in the drop tower experiment. Data of all experiments was combined and aligned to the impact timepoint. A histogram of the 300 s directly after the impact with 1 s bin size was generated. Firing rate directly after the impact is increased, decaying to a baseline level after about 10-20 s.
